# Genomic and physiological responses to strong selective pressure during late organogenesis: few gene expression changes found despite striking morphological differences

**DOI:** 10.1186/1471-2164-14-779

**Published:** 2013-11-11

**Authors:** Goran Bozinovic, Tim L Sit, Richard Di Giulio, Lauren F Wills, Marjorie F Oleksiak

**Affiliations:** 1 Department of Environmental and Molecular Toxicology, North Carolina State University, Box 7633, Raleigh, NC 27695-7633, USA; 2 Department of Plant Pathology, North Carolina State University, Box 7342, Raleigh, NC 27695-7342, USA; 3 Nicholas School of the Environment, Duke University, A333 LSRC, Box 90328, Durham, NC 27708, USA; 4 Rosenstiel School of Marine and Atmospheric Sciences, University of Miami, 4600 Rickenbacker Causeway, Miami, FL 33149, USA; 5 Current Address: Division of Biological Sciences, University of California at San Diego, HSS 1145G, 9500 Gilman Drive, La Jolla, CA 92093, USA

**Keywords:** Development, Genomics, Embryos, Adaptation, Evolution, Fish, Transcriptomics

## Abstract

**Background:**

Adaptations to a new environment, such as a polluted one, often involve large modifications of the existing phenotypes. Changes in gene expression and regulation during critical developmental stages may explain these phenotypic changes. Embryos from a population of the teleost fish, *Fundulus heteroclitus*, inhabiting a clean estuary do not survive when exposed to sediment extract from a site highly contaminated with polycyclic aromatic hydrocarbons (PAHs) while embryos derived from a population inhabiting a PAH polluted estuary are remarkably resistant to the polluted sediment extract. We exposed embryos from these two populations to surrogate model PAHs and analyzed changes in gene expression, morphology, and cardiac physiology in order to better understand sensitivity and adaptive resistance mechanisms mediating PAH exposure during development.

**Results:**

The synergistic effects of two model PAHs, an aryl hydrocarbon receptor (AHR) agonist (β-naphthoflavone) and a cytochrome P4501A (CYP1A) inhibitor (α-naphthoflavone), caused significant developmental delays, impaired cardiac function, severe morphological alterations and failure to hatch, leading to the deaths of reference embryos; resistant embryos were mostly unaffected. Unexpectedly, patterns of gene expression among normal and moderately deformed embryos were similar, and only severely deformed embryos showed a contrasting pattern of gene expression. Given the drastic morphological differences between reference and resistant embryos, a surprisingly low percentage of genes, 2.24% of 6,754 analyzed, show statistically significant differences in transcript levels during late organogenesis between the two embryo populations.

**Conclusions:**

Our study demonstrates important contrasts in responses between reference and resistant natural embryo populations to synergistic effects of surrogate model PAHs that may be important in adaptive mechanisms mediating PAH effects during fish embryo development. These results suggest that statistically significant changes in gene expression of relatively few genes contribute to the phenotypic changes and large morphological differences exhibited by reference and resistant populations upon exposure to PAH pollutants. By correlating cardiac physiology and morphology with changes in gene expression patterns of reference and resistant embryos, we provide additional evidence for acquired resistance among embryos whose parents live at heavily contaminated sites.

## Background

We studied genomic responses to the effects of chemicals routinely found in complex mixtures of pollutants present in the urban estuaries during late organogenesis of *Fundulus heteroclitus* embryos. Natural *Fundulus* populations are one of the few studied fish species in North America that have established resistant populations in highly contaminated urban estuaries [1]. Changes in gene expression, coupled with biochemical, physiological, and behavioral alterations play a critical role in adaptation to environmental stress. Our study explores the ways natural populations may have adapted to local polluted environments by correlating their genomic responses to changes in morphology and physiology during development.

*Fundulus heteroclitus*, a small, abundant, salt marsh fish that inhabits the eastern North American coast, has become a leading model in environmental biology. Natural *Fundulus* populations can tolerate a variety of environmental conditions and display an array of adaptations to both natural and anthropogenic variables in their ecosystems. *F. heteroclitus* is an ecologically important and genetically diverse model to elucidate pollution effects and genotype–environment interactions within and among natural populations.

*F. heteroclitus* is one of the few studied species in North America living in the highly polluted urban estuaries that has shown resistance to pollutants among both adults and embryos [2-5]. Multiple *Fundulus* populations inhabit and have adapted to heavily contaminated urban estuaries [4,6,7] which contain persistent and bioaccumulative chemicals that are toxic to early fish development [2,4]. Acute and chronic exposure of *Fundulus* embryos to chemicals present in the polluted sites can lead to functional deficit, growth retardation, malformation, and even death [3,4,6].

Resistance to the lethal effects of pollution has been reported in *F. heteroclitus* embryos from the Elizabeth River, VA, a Superfund site contaminated with creosote, a mixture of polycyclic aromatic hydrocarbons (PAHs) [7,8]. PAHs are petroleum products created by the combustion of organic materials that originate from both natural and anthropogenic sources. They have been found at 600 of 1,430 National Priority List sites, and as a group they are ranked number eight on the 2007 Comprehensive Environmental Response, Compensation, and Liability Act (CERCLA) priority list of hazardous substances [9,10]. These chemicals pose a significant risk to human and animal health due to their carcinogenic properties; research in aquatic organisms has described their equally damaging role as teratogens [11,12]. The PAH concentrations in the sediments collected at the Elizabeth River site are some of the highest in the world [8], averaging 200–400 ug/g. These PAHs include carcinogens, such as benzo(a)pyrene (BaP), chrysene, and dibenzo(a,h) anthracene [13,14].

Cardiovascular malformations, resulting in significantly higher mortality rates, are well documented in fishes exposed to PAH mixtures [12,15-18]. Some of these effects are thought to be mediated by the aryl hydrocarbon receptor (AHR) [15,19-22]. Studies of cardiovascular effects using model PAHs show synergistic interaction between PAH-type AHR agonists and CYP1A-inhibitors. Typically, the AHR pathway is induced by PAHs and activates expression of CYP1A [23]. In *F. heteroclitus*, embryos exposed to the PAH-type AHR agonist β-naphthoflavone (BNF) and the CYP1A inhibitor α-naphthoflavone (ANF) had decreased levels of CYP1A activity and a synergistic increase in the occurrence of cardiac deformities as measured by heart elongation and pericardial edema [17]. BNF and ANF are synthetic flavonoids commonly used as surrogate model PAHs: BNF acts as an AHR agonist and ANF acts as a reversible competitive CYP1A inhibitor that can bind to either the active site or the ferric heme [24-26]. Agonists and inhibitors often co-occur in typical PAH mixtures, and although the current risk assessments of PAHs assumes an additive model of PAH toxicity, this synergy may be an important outcome for risks posed by PAH-exposure.

*Fundulus* gene-environment interaction studies include both natural and anthropogenic environmental effects on anatomy, physiology, development, molecular biology, and recently a genome [27-30]. Consequently, natural *Fundulus* populations have become a preferred model to study teleost evolutionary adaptations to a range of selective pressures. The effects of pollution on *Fundulus’* genetics have been studied in some populations [1,31-36]; however, little is known about functionally important variation in embryo gene expression underlying resistance mechanisms. To explore the response differences between reference and resistant populations to pollution at the genomic and molecular level, we exposed embryos of parents from King’s Creek, VA (reference) and Elizabeth River, VA (resistant) to a defined surrogate mixture of PAH pollutants, which are routinely found in the contaminated sediment extracts. By correlating multiple phenotypes to changes in gene expression patterns, we provide additional evidence for acquired resistance among embryos whose parents live at heavily contaminated sites.

We chose to study late organogenesis of *Fundulus* development due to its high metabolic activity, observable phenotypes, and likely cumulative effect of chemical exposure on organ anatomy and physiology. Hence, we compare survival rates, time-to-stage, morphology, cardiac physiology, and gene expression profiles of individual *Fundulus* embryos from PAH-reference and resistant natural populations exposed to a mixture of two model PAHs at environmentally relevant concentrations [14]. Our study demonstrates important contrasts in responses between reference and resistant embryos to synergistic effects of this defined mixture of pollutants. Altered phenotypes and significant changes in gene expression reveal evidence for acquired resistance among embryos from heavily contaminated sites. However, while the phenotypic alterations are comparable to embryo responses to polluted sediment extracts (unpublished data), a surprisingly few number of significant genes reflect differences in severity of synergistic effects between the two embryo populations.

## Results

### Embryo survival, hatching success, and developmental delays

**Figure 1:**
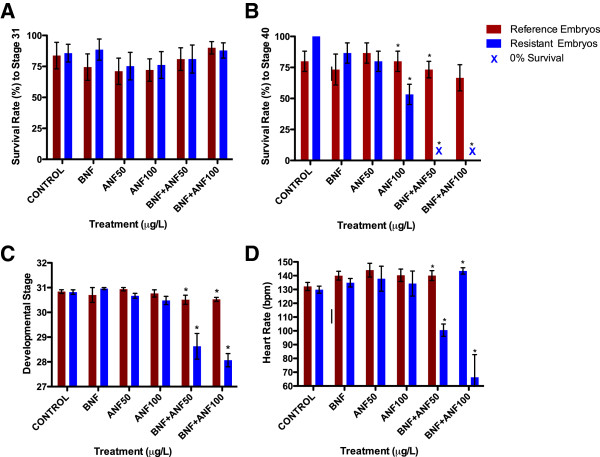
**Survival, developmental delays, and heart rates of reference (Kings’ Creek – blue) and resistant (Elizabeth River – red*) Fundulus *embryos. A)** Embryo survival (2-way ANOVA, p = 0.97) among five treatment groups within reference and resistant embryo populations at developmental stage 31 (late organogenesis). Sensitive and resistant embryo’s survival is not affected by any treatment at the stage 31*.***B)** Hatching success and survival to stage 40 (2-way ANOVA, p < 0.01) among reference and resistant embryos. The cumulative effects in both combined treatments have significant effect among sensitive embryos, as they fail to hatch, while the resistant embryo survival is largely unaffected. **C)** Development among control and five treatment groups of reference and resistant embryos at 144–150 hours post-fertilization: although most of the embryos reached stage 31 within the expected time period, significant developmental delays were noted among reference embryos exposed to both low and high ANF + BNF treatments (2-way ANOVA, P <0.01). Reference embryos were significantly delayed (on average 3 developmental stages, ~ 40 h) relative to resistant embryos in both combined treatments (Bonferroni post-test analysis, p < 0.01), while embryo development in discrete treatments did not significantly differ. **D)** Embryo heart rates: reference and resistant embryos’ heart rates during stage 31: significant bradycardia (2-way ANOVA, p < 0.01) were noted among reference embryos at both lower (p < 0.04; t = 4.12) and higher (p < 0.05; t = 8.03) BNF/ANF dose exposures relative to resistant embryos. Asterisks (*) represent statistically significant within-treatment differences (Student’s t-test, p < 0.05) between Elizabeth River (resistant) and King’s Creek (reference) embryos.

During *Fundulus* late organogenesis (stage 31), survival rates were not significantly different between populations for all treatment groups (2-way ANOVA, p = 0.97; Figure 1A. The lowest survival was noted among reference embryos treated with low ANF (50 ug/L, 71.1% ±10.7), while the highest survival rate was among resistant embryos (90% ± 5.0) treated with BNF (1 ug/L) + high ANF (100 ug/L). All of the embryos that hatched survived to the final embryonic stage characterized by complete yolk consumption and free-swimming larvae.

At stage 40, all of the reference embryos failed to hatch and did not survive either the lower or higher ANF/BNF co-exposure (Figure 1B). A 2-way ANOVA (p < 0.05) indicated significant differences in hatching success among embryo treatment groups. Bonferroni’s post-test revealed significant differences between reference and resistant embryos for both BNF + low ANF (p < 0.001, t = 6.74), and BNF + high ANF (p < 0.001, t = 6.12) treatment groups.

Although most of the embryos reached stage 31 within the expected time period, significant developmental delays were noted among reference embryos exposed to both low and high ANF + BNF treatments (2-way ANOVA, P <0.01; Figure 1C). Bonferroni post-test analysis of developmental stage at 144–150 hours post fertilization revealed significant differences among reference embryos and between reference and resistant embryos in both combined treatments (p < 0.01), while embryo development in discrete treatments did not significantly differ. Reference embryos were on average 3 stages behind (approximately 40 hours) when compared to resistant embryos from the same combined treatment group (2-tailed t-test, p < 0.01).

### Heart rate

Heart rate results at stage 31 mirrored developmental delay data: significantly slower heart rates (bradycardia; 2 way-ANOVA, p < 0.01; Figure 1D) were noted among sensitive embryos in combined treatment groups. Bonferroni post-test revealed statistically significant differences in reference embryos exposed to BNF + low ANF (100.5 ± 10.1 bpm; p < 0.01; t = 4.12) and BNF + high ANF (66.3 ± 36.8 bpm; p < 0.01; t =8.03) when compared to all other treatment groups in both reference and resistant embryo populations.

### Embryo morphology

**Figure 2:**
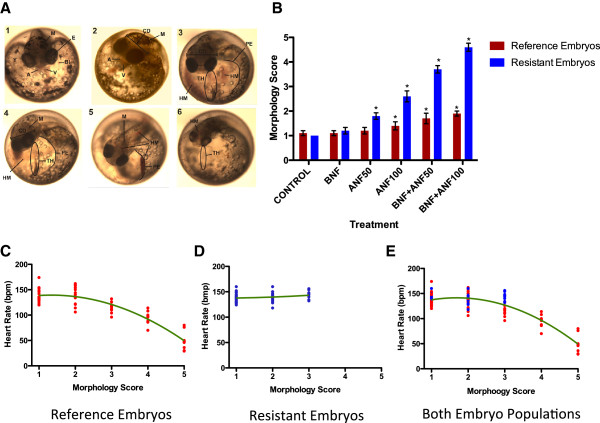
**Morphology, morphology scores, and heart rate – morphology correlations of reference and resistant embryos. A)** Embryo Morphology: progression of deformities among exposed reference embryos: 1 = control embryo; 2 = 1 μg/L BNF –exposed embryo; 3 = 50 μg/L ANF – exposed embryo; 4 = 100 μg/L ANF – exposed embryo; 5 = 1 μg /L BNF + 50 μg/L ANF – exposed embryo; 6 = 1 μg/L BNF + 100 μg/L ANF – exposed embryo. A – atrium; BI – blood island; E – eye; CD – cranial deformity (reduced cranial width and eye distance, diminished cranial ridges); HM – hemorrhage; M – melanin; PE – pericardial edema; TH – tube heart; V – ventricle; **B)** Embryo deformity assessment among reference (blue) and resistant (red) embryos under 6 treatments. 2-way ANOVA (p < 0.010 and Bonferroni post-test (p < 0.01) revealed statistical differences in 4/6 treatments between embryo populations. Asterisks (*) represent statistically significant within-treatment differences (Bonferroni post-test, p < 0.05) between Elizabeth River (ER - resistant) and King’s Creek (KC - reference) embryos. **C)** Correlation between Embryo Morphology and Heart Rate: **A)** Strong correlation (R^2^ = 0.82) is apparent among reference embryos; **B)** No correlation is apparent among resistant embryos (R^2^ = 0.044); **C)** Combined data of reference and resistant embryos shows strong correlation between progression of deformities and decrease in heart rates (R^2^ = 0.78).

Severe and extreme morphological abnormalities were noted among all reference embryos in combined treatment groups (Figure 2B). These deformities included pericardial edema, hemorrhaging, cranio-facial malformations, tail shortening and bleeding, and general loss of pigment (Figure 2A). The most severely affected reference embryos in BNF + high ANF treatments were characterized by overall smaller size, loss of cranial ridges, cranium size reduction with diminished eye distance, aggregation and reduction of body pigmentation and hemorrhaging throughout the entire caudal region; these morphologies were only observed among the reference embryos in higher co-exposure treatment group. Their hearts (score > 4 on a scale of 1–5 where 1 indicates no deformity and 5 indicates extreme deformity) failed to differentiate, resulting in a “tube-heart” structure, which appears as a barely-visible long tube through which transparent fluid slowly trickles (instead of 2 fully-formed round chambers with red blood forcefully pumping from the atrium into a ventricle, as seen in the control embryos). The average score for the BNF + low ANF treatment group was 3.7 and was 4.6 for the BNF + high ANF treatment group. Any embryo with a score > 3 failed to hatch. Statistical differences (Bonferroni post-test, p < 0.05) were noted between reference and resistant embryos in 4/6 treatments, with reference embryos being significantly more deformed at low ANF (p < 0.05; t = 2.91), high ANF (p < 0.01; t = 5.827), BNF + low ANF (p < 0.01, t = 9.71), and BNF + high ANF (p < 0.01, t = 13.11).

The relationship between heart rates and *in vivo* morphological deformities is presented in Figure 2C-E. Combined data of reference and resistant embryos show a strong correlation between heart rate and morphology (R^2^ = 0.78; Figure 2E): as the deformities progress among reference embryos throughout treatments, the bradycardia becomes more pronounced, reflecting the impaired heart function among reference embryos. A similar trend is apparent among reference embryos only (Figure 2D) showing the strong correlation (R^2^ = 0.82) between the progression of deformities and bradycardia among reference embryos. However, this is not the case for resistant embryos (Figure 2E), as progression of deformities does not correlate with the decrease in heart rate (R^2^ = 0.044). Moreover, the resistant embryos show a slight increase in heart rates (tachycardia) as deformities progress. Notably, none of the exposed resistant embryos were scored higher than 3 (moderate deformities) while all of the reference embryos in co-exposures with BNF and ANF were scored between 4 (severe deformities) and 5 (extreme deformities).

### Gene expression

**Figure 3:**
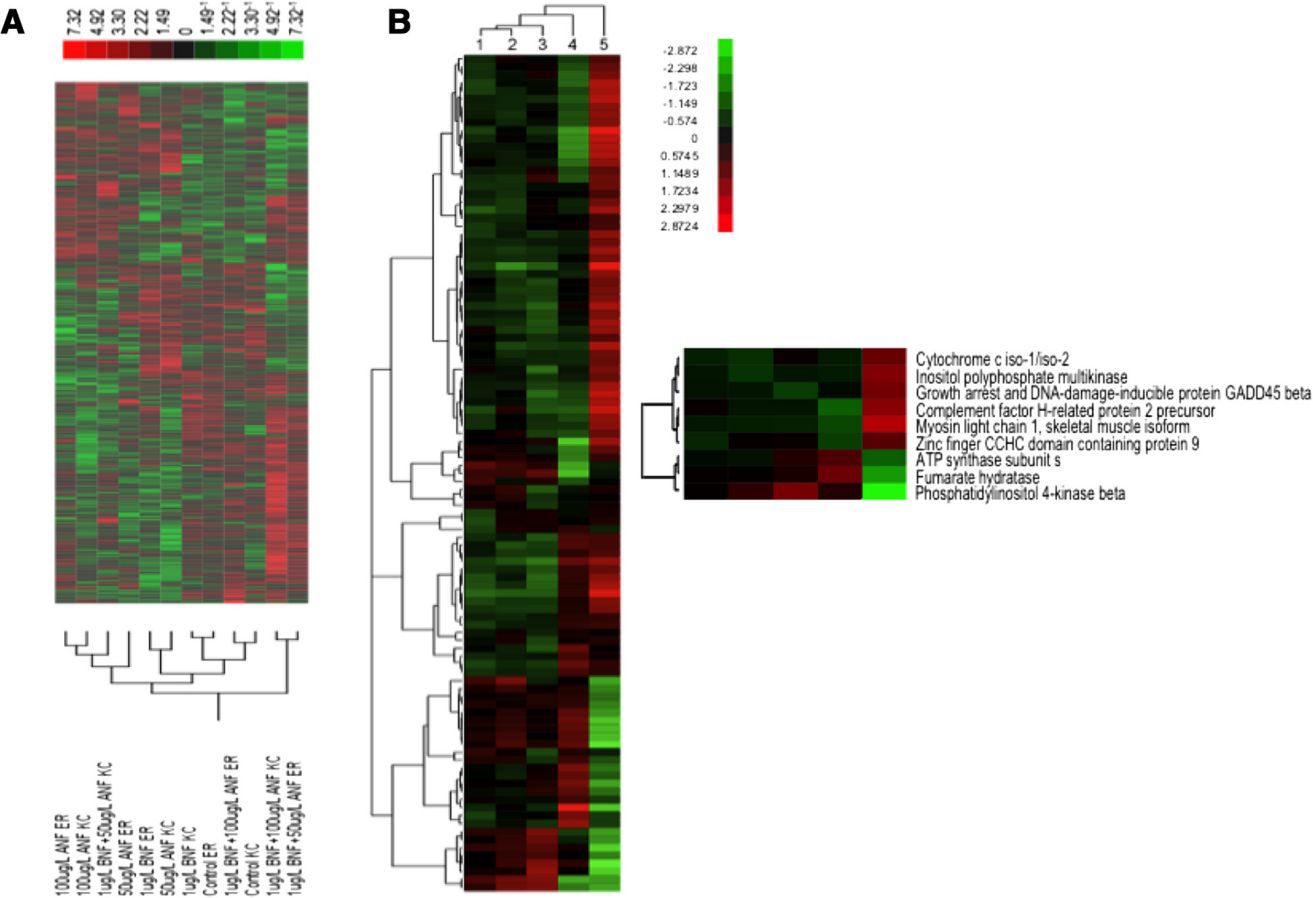
**Gene expression of reference and resistant embryos exposed to PAH-surrogate chemicals. A)** Heat map of significant genes reflecting all pairwise comparisons (p < 0.01) between reference and resistant embryos. Hierarchical clustering of the treatments (gene tree) is shown on the bottom of the heat map. Red indicates relative high expression levels and green represents low expression levels. **B)** Expression of 105 genes differs significantly (Mixed model ANOVA, p < 0.01) due to the effect of embryo morphology among reference and resistant embryos. Genes are clustered based on morphology scores, shown across the top of the heat map. Embryo morphology score was based on a 1–5 scale, 1 representing no deformities, 2-mild, 3-moderate, 4-severe, and 5-extreme, respectively. Subset of genes differentially expressed among severely deformed embryos relative to other treatment groups is shown to the right of the heat map. Red indicates high expression levels and green represents low expression levels.

**Figure 4:**
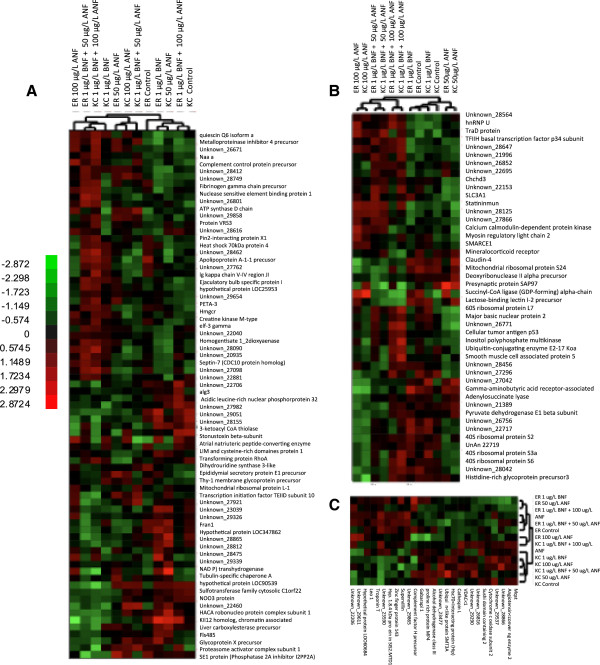
**Heat maps of differentially expressed genes due to population-by-treatment, treatment, and population effects among the reference and resistant embryos exposed to PAH-surrogate chemicals (Mixed model analysis ANOVA, p < 0.01). A)** Expression patterns of 73 significant genes in population-by-treatment effect; **B)** 52 genes are significantly different due to a treatment effect; **C)** 26 genes are significantly different due to differences between reference and resistant embryo populations. Hierarchical clustering of genes based on the treatment is shown across the top of the heat maps. Red color indicates relative high expression levels and green represents low expression levels.

Of the 6,754 genes analyzed for altered expression patterns, expression of 151 genes (2.24%) is significantly different (mixed model ANOVA, p < 0.01; Figure 3A). The combined effect of population and treatment analysis reveals 73 significant genes (Figure 4A). Expression of 52 genes is significantly different due to the effect of treatment alone (Figure 4B), while 26 genes are differentially expressed due to differences between reference and resistant embryo populations (Figure 4C). We found no significant genes when a False Discovery Rate (FDR) correction is applied at p < 0.05.

Expression of 105 genes (1.6%) is significantly different (mixed model ANOVA, p < 0.01) due to the effects of embryo morphology among reference and resistant embryos (Figure 3B). While gene expression appears similar among embryos scored for no deformities (index 1), mild (index 2) and moderate (index 3), the most differences in gene expression patterns are among severely deformed (index 4) and extremely deformed (index 5) embryos. Notably, all of the reference embryos exposed to BNF + low ANF and BNF + high ANF were found to be either severely or extremely deformed, while none of the resistant embryos were found to be more than moderately deformed in any treatment.

## Discussion

Increased mortality rates among adults and embryos, decreased fecundity, and impairment of physiological performances are likely effects of chronic exposure to pollution among populations [37-40]. Such effects may lead to a decrease in effective population size and genetic variability [18]. However, individual responses vary, and while some individuals are sensitive to the pollutants, others survive and reproduce, therefore establishing resistant populations. This acquired resistance is associated with fitness costs so that resistance genes are rarely fixed in natural populations [41]; counterbalancing selection pressure decreases the frequency of resistance genes in the absence of inducer, such as a chemical pollutant.

Embryos are highly sensitive to pollution and exposure to contaminated water and sediments can result in altered development and growth and can affect survival. When exposed to highly polluted sediment extracts, *F. heteroclitus* embryos from reference sites show significantly higher numbers of developmental abnormalities and do not survive, while most of the embryos from polluted sites are resistant and develop normally [3,4,6,7]. What changes contribute to this resistance? Adaptation to a new environment, such as a polluted one, often involves large modifications of the previous phenotype(s) and changes in gene expression and regulation during critical developmental stages may explain these phenotypic changes. Notably, altered gene regulation can affect development, resulting in different morphological or physiological characteristics [42] that are potentially critical for developing resistance.

### Differences in survival and development between reference and resistant embryos

Our study demonstrates important differences between reference and resistant embryo responses during one distinct developmental stage (late organogenesis) to a defined mixture of pollutants found in the sediment extracts. We exposed reference and resistant embryos to surrogate model PAHs to better understand physiological, morphological, and gene expression changes underlying development in a polluted environment. By correlating multiple phenotypes to changes in gene expression patterns, we provide additional evidence for acquired resistance among embryos whose parents live at heavily contaminated sites.

Combined treatments of β-naphthoflavone (BNF) and α-naphthoflavone (ANF) were lethal to the reference embryos, while the resistant embryos were largely unaffected: all of the reference embryos failed to hatch and died, while 70% of the resistant embryos reached the free-swimming larval stage (Figure 1A-B)*.* In all of the phenotypes assessed – survival, developmental delays, cardiac physiology (heart rate), and embryo morphology, the reference embryos were significantly more affected than the resistant embryos: while most treatments caused very little effect on development of resistant embryos, the same exposures caused significant developmental delays, impaired cardiac function, severe morphological alterations and failure to hatch, ultimately causing the death of reference embryos.

Development of reference embryos was significantly delayed among reference embryos (Figure 1C) in the high ANF exposures and both BNF-ANF co-exposures, indicating embryotoxic effects of ANF alone [25,26] and in synergy with BNF. On average, reference embryos lagged almost two days (3 stages, approximately 40 hours) behind resistant embryos given the same exposure. Importantly, exposed resistant embryos developed within the expected time period of both resistant and reference control embryos.

### Effects of pollutants on morphology, cardiac anatomy, and physiology on reference and resistant embryos

Prior to hatching, reference embryos became severely and/or extremely deformed (Figures 2A and 2B), resulting in altered physiology evident by impaired cardiac performance (Figure 1D) and failure to hatch. Although the average heart rate increased slightly among co-exposed resistant embryos, the overall cardiac function did not statistically differ between reference and resistant control embryos. We noted the most profound abnormalities in cardiac morphology among reference embryos co-exposed to BNF and ANF: the heart chambers of these embryos failed to differentiate and ultimately resembled elongated transparent tubes (“tube hearts”) with very limited contracting ability. We observed significant bradycardia among reference embryos co-exposed to BNF and ANF when compared to control embryos of both populations and resistant embryos exposed to the same co-exposures. Other deformities included pericardial edema, severe hemorrhaging, tail shortening, cranio-facial shrinkage, reduced eye distance, and gross loss of pigmentation (Figure 2A). In a few cases, the extreme deformities among reference embryos made identifying structures difficult. In contrast, none of the resistant embryos co-exposed to BNF and ANF were more than moderately deformed. Most (95%) of the resistant embryos developed fully differentiated heart chambers, capable of delivering blood throughout the embryo. Abnormal morphologies among resistant embryos included slight cranio-facial alterations, loss of pigment, mild to moderate pericardial edema, and tail hemorrhaging. Importantly, overall cardiac function of exposed resistant embryos was not affected and did not significantly differ from both reference and resistant control embryos.

We report a strong correlation (82%) between the severity of morphological deformities and cardiac physiology (Figure 2C-E) among reference embryos. As heart rates significantly decrease and become inefficient in delivering blood to the tissues due to BNF-ANF co-exposures, reference embryos become severely and extremely deformed. Their malformed hearts are unable to support development and embryogenesis ceases before hatching. Such is not the case among resistant embryos, and there is no relationship between the morphology score and cardiac function: heart rates remain unaffected in all resistant embryo treatment groups, demonstrating the ability of resistant embryos to cope with the synergistic effects of BNF-ANF co-exposure.

### Gene expression differences between reference and resistant embryos

Among the differentially expressed genes in embryos from Elizabeth River and King’s Creek, expression of 52 genes differs significantly due to treatment alone (Figure 4B), and hierarchical clustering of these genes groups most treatments of reference and resistant embryos together. Expression of 26 genes differs significantly because of differences between reference and resistant embryo populations (Figure 4C). Although our previous comparison of five independent *Fundulus* populations using the same arrays revealed 30 genes that significantly differ between King’s Creek and Elizabeth River embryos at stage 31 [43], there is no gene overlap with this data. This lack of overlap may reflect the fact that the current comparison examined embryos treated with PAHs while the previous experiment examined untreated embryos collected from parents from King’s Creek and the Elizabeth River. Some of the changes in gene expression noted in our present study may be simply due to changes in embryo morphology due to specific chemical exposures, which do not represent other pollutants present in the complex mixtures found in heavily contaminated sites. Also, the spatial and temporal variation associated with chemical exposure among natural populations in the wild may contribute to changes in gene expression not observed in our study.

Expression of 72 genes differs significantly due to the treatment-by-population interaction (Figure 4A). These expression patterns reveal similarities between reference embryos exposed to the higher synergy treatment and resistant embryos exposed to the lower synergy treatment. Interestingly, resistant embryos treated with the highest co-exposure of BNF and ANF group with reference and resistant controls and reference embryos exposed to low ANF, while exposure to BNF alone does not seem to be a determining factor in the cluster analysis.

There is a striking relationship between the morphology score and differential expression of genes (Figure 3B). As synergistic treatment concentrations increase among reference embryos, the severity of deformities observed among embryos increases, causing significant overall reduction in embryo size, bradycardia, disproportional size reduction of cranium including diminished distance between eyes, complete loss of cranial ridges, reduction of eye and body pigment, hemorrhaging along the entire shortened caudal region, cardiac edema, and complete loss of cardiac muscle integrity characterized by the absence of heart chambers and formation of a thin-walled, translucent “tube heart”. Expression patterns of genes that correlate with morphology are similar among normal to moderately deformed embryos (1–3 on morphology score scale), while severely deformed embryos (score of 4) show different patterns of gene expression. Gene expression differences become more pronounced between extremely deformed embryos when compared to normal to moderately deformed and severely deformed embryos. Importantly, only reference embryos were scored >3 in both lower and higher BNF-ANF co-exposure treatments, providing further evidence of PAH-resistance in the Elizabeth River (resistant) embryo population.

Several genes listed in Table 1, whose expression is correlated with observed morphological abnormalities, are known to play an important role during organogenesis. Although most differences in expression are between 1.4-2.3 fold, relatively small changes in transcript levels may contribute to the morphological and physiological alterations observed among developing embryos. For example, cytochrome C oxidase iso-1/iso-2 and complement factor H-related protein 2 have 1.51 fold and 1.43 fold higher transcript levels, respectively, among severely deformed reference embryos in BNF + high ANF treatment group. Both genes are linked to cardiovascular deformities of Libman-Sacks endocarditis and antiphospholipid syndrome (aPLs), marked by mitral and aortic valve lesions. Such abnormalities can cause severe valvular insufficiency, infective endocarditis, stroke [44] and cerebrovascular complications [45]. We noted severe morphological alterations in cardiac tissue in the form of a “tube heart” [28], with significant bradycardia among reference embryos co-exposed to BNF and ANF, suggesting that differences in expression levels of these two genes among both reference and resistant embryo populations may contribute to their cardiac deformities. Myosin light chain isoform 1 (ELC/RLC) and growth arrest and DNA damage-inducible protein GADD45 beta gene are upregulated (>7.3 fold and 3.3 fold, respectively) in severely deformed reference embryos relative to all other treatment groups in both embryo populations (Figure 3B). ELC/RLC overexpression leads to increase in cardiomyocyte size and number resulting in large ventricular chamber volume [46]. Relatively higher expression of these genes may explain severe cardiac abnormalities observed in reference embryos caused by synergistic effect of BNF and ANF in BNF + high ANF treatment group. Moreover, both knockdown and over expression of GADD45 beta genes cause somite defects with different consequences for marker gene expression, suggesting that regulated expression of GADD45 beta genes in the anterior PSM is required for somite segmentation [47]. Overexpression (>3.3 fold; Figure 3B) of GADD45 in severely deformed reference embryos may contribute to synergistic effects if BNF + high ANF treatment and contribute to skeleto-muscular abnormalities linked to heart abnormalities during late embryogenesis.

**Table 1:** **Subset of embryogenesis-related genes whose expression significantly differs between reference (King’s Creek) and resistant (Elizabeth River)** ***Fundulus heteroclitus*** **embryos (Mixed model ANOVA, p < 0.01) during late organogenesis stage due to the effect of embryo morphology**

**Gene**	**Fold diff.**	**Effect**	**Ref**
Cytochrome C oxidase iso-1/iso-2	1.51	Libman-Sacks endocarditis and antiphospholipid syndrome (aPLs)	[44,45]
Complement factor H-related protein 2	1.43	Libman-Sacks endocarditis and antiphospholipid syndrome (aPLs)	[44,45]
Myosin light chain (ELC / RLC)	1.94/1.3	Increase in cardiomyocyte size and number, resulting in a larger ventricular chamber volume/reduction in size and number of cardiomyocytes	[48,49]
ATP synthase subunit S	1.47	Cellular ATP synthesis; depletion of the cellular ATP pool during ischemia	[46,50]
GADD45 beta	1.56	Cell-cycle control; Somitogenesis abnormalities	[47]
Inositol polyphosphate multikinase (IPMK)	1.66	mRNA export, transcriptional regulation, and chromatin remodeling	[51]
Phosphatidylinositol phosphate kinase 4 (PIPK) beta	2.25	Mouse embryo brain expression (cerebral ventricular and mantle zones) during normal development; postnatal brain gray matter expression	[47]
Fumarate hydratase (fumarase)	1.77	Severe neurologic fetal brain abnormalities, poor feeding, failure to thrive; hypotonia, severe mental retardation, unusual facial features, brain malformation, epileptic seizures	[52,53]

The expression pattern of genes for each morphology score group is presented in Figure 3b. While gene expression appears similar among embryos scored for no deformities (score 1), mild (score 2) and moderate (score 3), most differences in gene expression patterns are among severely deformed (score 4) and extremely deformed (score 5) embryos. Notably, all of the reference embryos co-exposed to ANF and BNF were either severely or extremely deformed, while none of the resistant embryos were found to be more than moderately deformed in any treatment.

Several other genes whose significant changes in expression correlate to morphology are implicated in metabolism and CNS development. The ATP synthase subunit S gene, which is >4 fold overexpressed in severely deformed reference embryos relative to moderately-deformed reference embryos (Figure 3B) is a critical enzyme in the cell’s energetic pathways, producing the majority of cellular ATP and energetics of the heart which are integrally involved in the causes and phenotypes of heart failure [54,55]. Inositol polyphosphate multikinase (IPMK) plays a critical role in nuclear functions including mRNA export, transcriptional regulation, and chromatin remodeling. Ipk-2-deficient mice die around embryonic day 9.5 with multiple morphological defects, including abnormal folding of the neural tube [51]. IPMK displays a similar overexpression pattern as ELC/RLC and GADD45 in severely deformed reference embryos, likely contributing to observed severe morphological abnormalities among reference embryos exposed to ANF + high BNF treatment.

Notably, significantly lower expression of two genes among reference embryos exposed to BNF + high ANF treatment may contribute to severe morphological deformities. Phosphatidylinositol phosphate kinase 4 (PIPK) beta, which is expressed in the mouse embryo brain (cerebral ventricular and mantle zones), plays a role in the formation of cerebral ventricular and mantle zones and gray matter during normal development [47]. Deficiency in fumarate hydratase (fumarase), a gene expressed in human fetal tissues [56] is linked to a fetal brain and severe neurologic abnormalities, poor feeding, failure to thrive, hypotonia, encephalopathy [52], severe mental retardation, unusual facial features, brain malformation, and epileptic seizures [53]. We noted significant reduction in head size and complete loss of cranial ridges in severely deformed reference embryos.

Due to severe morphological abnormalities oberved among reference embryos, it was often difficult to accurately stage the embryos, which likely confounded some of our gene expression analyses. Significant changes in gene expression that corelate with morphology are similar among normal to moderately deformed embryos, while severely deformed embryos show different patterns of gene expression (Figure 3B). Moreover, the gene expression differences become more pronounced between extremely deformed embryos, when compared to both normal to moderately deformed and severely deformed embryos. Importantly, synergistic effects of BNF and ANF were only evident among severely and extremely deformed reference embryos, providing further evidence of PAH-resistance in the Elizabeth River (resistant) embryo population.

### Few genes contribute to large phenotypic changes between embryo populations

Despite the striking differences in embryo morphology between reference and resistant embryos, a relatively low percentage of genes (2.24% of 6,764) showed statistically significant differences in transcript levels (Figure 3A). This percentage is less than the percentage of genes significantly differentially expressed between PCB treated embryos from another resistant *Fundulus* population and a nearby reference site. At 15 days post-fertilization, 2.4% of genes differ with an FDR p-value of <0.01 between embryos from New Bedford Harbor, which are resistant to PCBs, and embryos from a reference population treated with PCBs [57]. Previous results comparing untreated embryos from parents collected from polluted resistant and reference populations also found a surprisingly small number of significantly differently expressed genes (using the same microarray platform, 0.8% of the genes differed significantly between embryos collected from parents from the Elizabeth River population and nearby reference populations [43]). One possible explanation for this finding was that differences would only manifest in the correct environment (e.g., gene by environment interactions). Given the data presented here, this explanation appears not to be true (at least for the combined treatment of BNF and ANF). Considering the large variation in gene expression reported within and among multiple reference and resistant adult *F. heteroclitus* populations [27,28,58-60], this low percentage of genes whose expression significantly differs between both treated and untreated reference and resistant embryo populations is unexpected.

This small percentage of significantly differentially expressed genes may be due to several factors. Relatively small changes in gene expression not detected by our analysis may be biologically important during late organogenesis. We did not test all of the genes expressed during development, so some of the important gene expression differences were likely missed. Also, some of the significant gene expression differences may be masked by large changes in gene expression that occur between stages during normal *Fundulus* development [61]. Our analysis was performed on whole embryos, thus potentially masking some tissue-specific gene expression differences. Critical differences in gene expression may occur at earlier or later developmental stages than the one we examined (stage 31, late organogenesis). However, a recent transcriptome comparison of PCB-exposed reference and resistant *Fundulus* embryos at two time-points during embryogenesis [5 and 10 days post fertilization (dpf), approximately stage 28–29, and 35, respectively] and one larval stage (15 dpf, approximately stage 39–40) revealed a stage-specific response and cumulative pollutant effect reflected by the increase of significantly expressed genes at later stages [57]. Arguably, more robust tissue-specific changes in gene expression occur during early development, particularly during early CNS (stages 16–19) and cardiovascular organogenesis (stages 16–25).

Finally, increasing a relatively small biological sample size per treatment (N = 4) and statistical power in our microarray analysis could have revealed more statistically significant genes. Previous tissue-specific studies on *Fundulus* adults using only one more individual (five *versus* four) from these same populations have reported up to 40% of genes that differ due to treatment (population) [27,58]. However, our recently published study comparing eight resistant and twelve reference, untreated embryos during late organogenesis using the same microarray platform revealed less than 1% of significant differently expressed genes [43]. Although we identified significant changes in gene expression and correlated them with multiple phenotypes, other factors not considered in our study, such as post-translational modifications and changes in protein expression and enzyme activity are likely contributors to observed differences between resistant and reference embryo populations.

## Conclusions

Our study demonstrates important contrasts in responses between reference and resistant natural embryo populations to synergistic effects of surrogate model PAHs that may be important in adaptive mechanisms mediating PAH effects during fish embryo development. While the reference embryos become severely deformed and none survive ANF/BNF co-exposures, the absence of moderate and severe deformities, lack of significant changes in heart rates and developmental delays, and >70% survival among resistant embryos co-exposed with BNF and ANF relative to reference and resistant control embryos clearly demonstrates the resistant embryos’ ability to adapt and survive. By analyzing multiple phenotypes and linking them to gene expression patterns of reference and resistant embryos, we provide additional evidence for acquired resistance among embryos whose parents live at heavily contaminated sites: while most treatments caused very little effect on development of resistant embryos, synergistic effects of a PAH-type representative AHR agonist and CYP1A inducer caused developmental delays, impaired cardiac function, morphological alterations, and mortality of reference embryos. These phenotypes mirror embryo responses observed during exposure to complex mixtures of pollutants found in Elizabeth River sediment extracts, but in contrast to exposure to sediment extracts that significantly altered expression of many genes (20% of 6,551 genes, unpublished data), we found a surprisingly small percentage of significantly differentially expressed genes (2.24% of 6,764 genes) upon treatment with a mixture of two model PAHs, α-napthoflavone and β-napthoflavone. These results suggest that relatively few genes contribute to the large phenotypic changes seen among reference and resistant populations upon exposure to PAH pollutants.

## Methods

### Fish care

Adult *Fundulus* were collected from both a reference site at King’s Creek, near the mouth of the York River in Gloucester County, Virginia (37°17′52.4″N, 76°25′31.4″W) and from a contaminated site on the Elizabeth River in Portsmouth, Virginia (36°48′27.48″N, 76°17′35.77″W). Adult fish were depurated for 12 months in a recirculating system containing artificial seawater (ASW 25 ppt) prepared from Instant Ocean® (Mentor, OH). Fish were kept at 23-25°C on a photoperiod of 14:10 L:D and fed a daily diet of Tetramin® Tropical Fish Food (Tetra Systems, Blacksburg VA, USA) and newly hatched brine shrimp (Artemia, Brine Shrimp Direct, Ogden, UT). Killifish embryos were obtained from *in vitro* fertilization of pooled oocytes mixed with pooled milt from multiple males. Embryos were examined 24 hours post fertilization (hpf) for viability and placed individually into 20 mL glass scintillation vials with 10 mL of treatment solution.

### Chemicals and exposure

Dimethyl sulfoxide (DMSO), ANF, BNF and ethoxyresorufin were purchased from Sigma-Aldrich (St. Louis, MO). King’s Creek (reference) embryos and Elizabeth River (sensitive embryos) were exposed to the following treatments:

Treatment 1: DMSO / vehicle (Control)

Treatment 2: 1 ug/L BNF (BNF)

Treatment 3: 50 ug/L ANF (low ANF)

Treatment 4: 100 ug/L ANF (high ANF)

Treatment 5: 1 ug/L BNF + 50 ug/L ANF (BNF + low ANF)

Treatment 6: 1 ug/L BNF + 100 ug/L ANF (BNF + high ANF).

Individual embryos of parents from both populations were exposed to the treatment solution or to the DMSO vehicle control from 24 to 120 hpf (n = 20). In all of the treatment groups, DMSO concentration was maintained at less than 0.03%. At 120 hpf, embryos were removed from the dosing solution and placed into vials containing clean ASW.

### Embryo survival, developmental delays, and heart rate

Fertilization success and embryo progress were monitored daily by examining representative stages during pre-determined time periods [61,62] using a dissecting stereo microscope (Nikon SME1500, Japan). Stage progression, developmental delays, normal *versus* abnormal development, and mortality also were recorded. Unfertilized eggs, malformed and/or dead embryos were removed from the population, and times and stages of arrest and abnormal development were recorded accordingly. Survival rates were measured within each treatment in both populations. Embryos that successfully reached stage 31 were used for heart rate and gene expression analysis. Ten embryos from each treatment group were assessed for hatching success and survival to complete embryogenesis, marked with the total yolk consumption by the free-swimming *Fundulus*[61,62].

To determine developmental delays, ten embryos from each population were monitored in individual 20 ml scintillation vials. Identification of each stage was determined using a dissecting stereo microscope at 70-80X magnification. At approximately 144–150 hpf, when the embryos were expected to reach stage 31 [61,62], multiple images of developing embryos were captured with the Micropublisher 5.0 RTV Camera (QImaging). These images were catalogued, stored, and analyzed using QCaputre Pro imaging software. Each embryo was scored based on multiple morphological characteristics and assigned the appropriate developmental stage.

The same embryos used to determine developmental delays were used to determine heart rates during early organogenesis (stage 31). A beating heart is formed, with both chambers completely differentiated and in full view, by stage 31 and the heart rate can be accurately measured from that stage on. Embryo vials were labeled to assure that the heart rate was measured from the same embryo at both stages. Individual embryos were placed on a depression slide under the dissecting stereo microscope for 1 minute prior to taking heart rate measurements so that the stressed embryo could re-establish resting heart beat (most *Fundulus* embryos temporarily arrest their heart beat due to a sudden change of environment, such as transfer from the petri dish to a well-lit slide surface). The heart rate of each embryo was measured by counting the number of heartbeats for 30 seconds (preliminary results showed no change in the average heart beat when counts were taken at either 30 second or 1 minute intervals).

### Embryo morphology

At 168 hours-post-fertilization (hpf), ten embryos from each treatment were randomly selected and subjectively scored treatment-blind twice independently (N = 2) for morphological abnormalities using light microscopy. Embryos were scored for severity of heart deformities (tube heart), pericardial edema, hemorrhaging, cranio-facial alterations, tail shortening, and pigment loss. Embryo score was based on a 1–5 scale, 1 representing no deformities, 2-mild, 3-moderate, 4-severe, and 5-extreme, respectively. Non-deformed embryos appeared wrapped approximately 2/3 around the full circumference of the remaining yolk, and with clearly distinguishable cranial ridges, well-defined dark-pigmented eyes with visible retinas, dark and scattered body pigment, clearly distinct atrial and ventricular cardiac regions, absence of hemorrhaging, and the caudal region approximately 1/3 of the body length beginning at the bilobed urinary bladder [61]. The most severely affected embryos were characterized by overall smaller size, disproportional size reduction of cranium including diminished distance between eyes, complete loss of cranial ridges, reduction of eye pigmentation, near-complete aggregation and overall reduction of body pigmentation, hemorrhaging along the entire shortened caudal region, and complete loss of cardiac muscle integrity characterized by the absence of heart chambers and formation of a thin-walled, translucent “tube heart”.

Results for each treatment were represented as an average of the individual scores.

While all phenotypes were considered in determining the final score, the heart deformities were found to be the most reference and reliable endpoint used in deformity assessment.

These experiments were performed according to approved protocols (Institutional Animal Care and Use Committees, North Carolina State University and Duke University).

#### Survival, heart rate, developmental delays, and morphology statistical analysis

Differences in the survival, heart rate, developmental delays, and morphology, among two embryo populations and six treatments were analyzed with Prism Statistical Software. Data were normally distributed and were analyzed using one-way Analysis of Variance (1-way ANOVA, p < 0.05); pairwise t-test was used to test the differences of means between treatment groups, while Dunnett’s one-tailed t-test was used to evaluate differences between “reference” embryos and “resistant” embryos, respectively.

### Microarrays

Amplified cDNA sequences for 7,000 genes from *F. heteroclitus* cDNA libraries were spotted onto epoxide slides (Corning) using an inkjet printer (Aj100, ArrayJet, Scotland). Libraries were made from all 40 stages of *Fundulus* development, immediately post-hatch whole larvae, and adult tissues. Each slide contained four spatially separated arrays of ~7,000 spots (genes) including controls. Sequence information, annotation and gene ontology are available for *Fundulus* on the FunnyBase website http://www.ccs.miami.edu/cgi-bin/Fundulus/Fundulus_home.cgi.

#### Embryo RNA isolation, amplification, and labeling

Four individual embryos from each treatment at developmental stage 31 [62] were used for RNA isolation, labeling, and microarray hybridization. Embryo RNA was extracted using a TRIzol buffer (Invitrogen, Carlsbad, CA, USA) followed by purification using the Qiagen RNeasy Mini Kit (Qiagen Inc., Valencia, CA, USA).

Purified RNA was quantified with a spectrophotometer, and RNA quality was assessed by gel electrophoresis. RNA for hybridization was prepared by one round of amplification (aRNA) using Ambion’s Amino Allyl MessageAmp aRNA Kit to form copy template RNA by T7 amplification. Amino-allyl UTP was incorporated into targets during T7 transcription, and resulting amino-allyl aRNA was coupled to Cy3 and Cy5 dyes (GE Healthcare, Piscataway, NJ, USA).

Labeled aRNA samples (2 pmol dye/μl) were hybridized to slides in 10 μl of hybridization buffer [50% formamide buffer, 5x SSPE, 1% sodium dodecyl sulfate, 0.2 mg/ml bovine serum albumin, 1 mg/ml denatured salmon sperm DNA (Sigma), and 1 mg/ml RNase free poly(A) RNA (Sigma)] for 44 hours at 42°C. Slides were prepared for hybridization by blocking in 5% ethanolamine, 100 mM Tris pH 7.8, and 0.1% SDS added just before use for 30 minutes at room temperature, washed for one hour in 4x SSC, 0.1% SDS at 50°C, and then boiled for 2 minutes in distilled water to denature the cDNAs. Resulting 16 bit Tiff Images were quantified using ImaGene® (Biodiscovery, Inc.) spotfinding software. Controls and any gene that did not have at least one individual with a signal greater than the average signal from all herring sperm control spots (non-specific hybridization signal) plus one standard deviation were removed prior to statistical analysis. In total, 6,754 genes were analyzed.

#### Experimental design for microarrays

**Figure 5:**
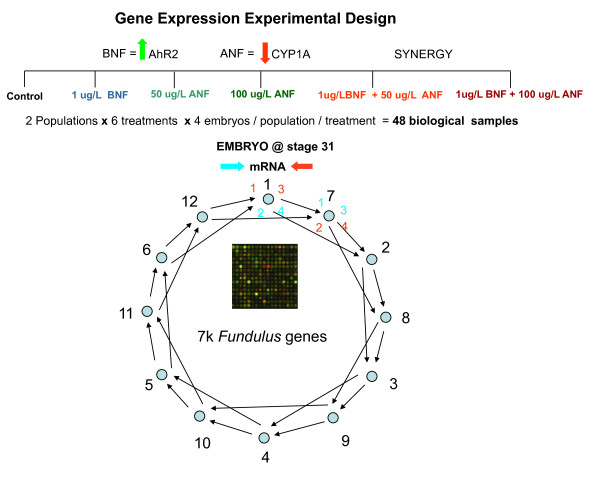
**BNF-ANF treatment exposures and microarray loop design: each sample is hybridized to 2 arrays using both Cy3 and Cy5 labeled fluorophores.** The loop consisted of Cy3 and Cy5 labeled embryo aRNAs from 4 biological samples and six different treatments (T1-T6: control, 1 μg/L BNF, 50 μg/L ANF, 100 μg/L ANF, 1 μg/L BNF + 50 μg/L ANF, 1 μg/L BNF + 100 μg/L ANF). Anticipated treatment effects on AHR and CYP450IA genes are shown with up and down green/red arrows above the treatments. 48 biological samples were hybridized to 24 microarrays. Each array had different combinations of biological samples, so that the most direct comparisons (*i.e.,* 50 μg/L ANF resistant embryo and 50 μg/L reference embryo) are hybridized to the same array. The loop formed was T1S → T1R → T2S → T2R → T3S → T3R → T4S → T4R → T5S → T5R → T6S → T6R → T1S → T2S → T3S → T4S → T5S → T6S → T1S → T1R → T2R → T3R → T4R → T5R → T6R, where each arrow represents a separate hybridization (array) with the biological sample at the base of the arrow labeled with Cy3 and the biological pool at the head of the arrow labeled with Cy5. T1-6 is treatment, and S and R represent reference and resistant embryos, respectively. A shorter example of the loop design shows treatments as numbers, and arrows as separate hybridization (array).

A loop design (Figure 5) was used for the microarray hybridizations where each sample is hybridized to 2 arrays using both Cy3 and Cy5 labeled fluorophores [63]. The loop consisted of Cy3 and Cy5 labeled embryo aRNAs from 4 biological samples and six different treatments (T1-T6: control, 1 μg/L BNF, 50 μg/L ANF, 100 μg/L ANF, 1 μg/L BNF + 50 μg/L ANF, 1 μg/L BNF + 100 μg/L ANF). In total, 48 biological samples were hybridized to 24 microarrays. Each array had different combinations of biological samples, so that the most direct comparisons (*i.e.,* 50 μg/L ANF resistant embryo and 50 μg/L reference embryo) are hybridized to the same array. The loop formed was T1S → T1R → T2S → T2R → T3S → T3R → T4S → T4R → T5S → T5R → T6S → T6R → T1S → T2S → T3S → T4S → T5S → T6S → T1S → T1R → T2R → T3R → T4R → T5R → T6R, where each arrow represents a separate hybridization (array) with the biological sample at the base of the arrow labeled with Cy3 and the biological pool at the head of the arrow labeled with Cy5. T1-6 is treatment, and S and R represent reference and resistant embryos.

#### Microarrays statistical analysis

Log_2_ measures of gene expression were normalized using a linear mixed model in SAS (JMP v6.0.0 with a microarray platform beta-version in SAS v9.1.3) to remove the effects of dye (fixed effect) and array (random effect) following a joint regional and spatial Lowess transformation in MAANOVA Version 0.98.8 for R to account for both intensity and spatial bias [64].

The model was of the form y_ij_ = μ + A_i_ + D_j_ + (AxD)_ij_ + ε_ij_, where, y_ij_ is the signal from the i^th^ array with dye j, μ is the sample mean, A_i_ and D_j_ are the overall variation in arrays and dyes (Cy3 and Cy5), (AxD)_ij_ is the array x dye interaction and ε_ij_ is the stochastic error [65,66].

Residuals from the above model were used for gene-by-gene analyses of treatment effect during a particular developmental stage, using treatment, population x treatment, and dye as fixed effects, and array and spot nested in array as random effects. The model was r_ijkng_ = μ + A_i_ + D_j_ + T_k_ + P_n_ + (TxP)_nk_ + ε_ijkn_ where *Tk* is the *k*^th^ treatment (treatments 1–6, above), *P*_*n*_ is the *n*^*th*^ population (reference or resistant), and (TxP)_nk_ is the treatment by population interaction. We also used residuals for a gene-by-gene analysis of morphology: r_ijk_ = μ + A_i_ + D_j_ + M_k_ + ε_ijk_ where *Mk* is the *k*^th^ morphology (morphology score 1 – 5 where 1 is normal and 5 is extremely deformed).

For all mixed model analyses, we used a nominal p-value cut-off for significant genes of p < 0.01. Using this p-value reveals more genes that may be differentially expressed but risks identifying genes that may be false positives.

Hierarchical clustering used JmpGenomics, Cluster 3.0 for Mac OS X, and Java TreeView version 1.0.8 [67].

### Data accessibility

Microarray data have been deposited in NCBI’s Gene Expression Omnibus (Edgar and Lash, 2002) and are accessible through GEO Series accession number GSE47654.

## Competing interests

The authors declare that they have no competing interests.

## Authors’ contributions

GB, RD, LW and MFO designed the experiment. GB and LW performed embryo exposures and developmental and morphological analysis. GB and TLS isolated and labeled embryo RNAs. GB and MFO performed hybridizations and statistical analyses of gene expression data and drafted the manuscript. All authors critically revised the manuscript and gave approval of the final version.
